# Free water as a potential mediator linking basal ganglia peri-vascular spaces to white matter hyperintensities in cerebral small vessel disease

**DOI:** 10.3389/fnins.2025.1621023

**Published:** 2025-07-03

**Authors:** Weishun Feng, Xinjun Lei, Xinbin Wang, Shida Xu, Zhihua Xu

**Affiliations:** ^1^Department of Radiology, Lishui Hospital of Traditional Chinese Medicine Affiliated Zhejiang Chinese Medical University, Lishui, China; ^2^Department of Radiology, The First People's Hospital of Xiaoshan District, Hangzhou, China; ^3^Department of Radiology, Tongde Hospital of Zhejiang Province, Hangzhou, China

**Keywords:** white matter hyperintensity, perivascular space, free water, interstitial fluid, cerebral small vessel disease

## Abstract

**Objective:**

White matter hyperintensities (WMH) are common in cerebral small vessel disease (CSVD) and have been linked to an increased risk of stroke and cognitive impairment. Emerging evidence suggests that perivascular spaces (PVS) and impaired interstitial fluid (ISF) drainage may contribute to WMH development. This study aimed to investigate the topographical association between PVS and WMH severity, and to explore whether ISF content, measured as diffusion tensor imaging (DTI)-derived free water (FW), mediates this relationship.

**Methods:**

We enrolled 125 patients with CSVD who underwent multimodal brain MRI. PVS burden was visually rated in the basal ganglia (BG PVS) and centrum semiovale (CS PVS). WMH volumes were segmented and normalized to intracranial volume. FW maps were generated from DTI using bi-tensor modeling.

**Results:**

Patients with high grade BG PVS exhibited significantly greater normalized WMH volumes compared to those without (*p* < 0.05), whereas no significant difference was found for CS PVS. Multivariable analysis indicated that high grade BG PVS was independently associated with increased WMH burden (β = 0.217, 95% CI: 0.041–0.393; *p* < 0.05). Mediation analysis demonstrated that FW mediated the association between high grade BG PVS and WMH severity (β = 0.082, 95% CI: 0.012–0.207; *p* < 0.05) adjusting with age and vascular risk factors.

**Conclusion:**

Our findings suggest a topographical specificity in the PVS-WMH relationship, with basal ganglia PVS playing a more critical role. Moreover, elevated ISF, as indicated by DTI-based FW, may be an important intermediary linking PVS enlargement to WMH burden, providing new insights into the pathophysiology of CSVD.

## 1 Introduction

White matter hyperintensities (WMH), frequently observed in cerebral small vessel disease (Duering et al., 2023), are mechanistically linked to elevated risks of cerebrovascular events and cognitive decline (Sun et al., 2025), posing a growing threat to public health. However, many of underlying factors that contribute to WMH remain unclear.

Among emerging contributors to WMH, perivascular spaces (PVS), fluid-filled channels surrounding small cerebral vessels, have drawn increasing attention (Huang et al., 2021; Barnes et al., 2022; Menze et al., 2024). Recent evidence suggests that PVS are critical anatomical structures in the glymphatic pathway that enable efficient interstitial fluid (ISF) circulation and removal of metabolic waste clearance (van Veluw and Perosa, 2022). PVS enlargement may reflect impaired glymphatic drainage, leading to ISF stagnation and the accumulation of neurotoxic substances, which in turn may contribute to white matter damage and WMH formation.

Importantly, the anatomical location appears to influence the pathological relevance of PVS. PVS in the basal ganglia (BG PVS) follow the course of deep perforating arteries (e.g., lenticulostriate arteries), which are more vulnerable to hypertensive arteriopathy and demonstrate stronger pulsatile dynamics than superficial arterioles (van den Kerkhof et al., 2023). Basal ganglia PVS may be more sensitive indicators of early ISF dysregulation, and thus exhibit a stronger spatial relationship with WMH than PVS in the centrum semiovale (CS PVS).

To further investigate the fluid-based mechanisms linking PVS to WMH, we employed free water (FW) imaging, a diffusion tensor imaging (DTI) derived metric estimating the fraction of freely diffusing water in the extracellular space. While FW increases can arise from various pathological processes (Qian et al., 2022)-including neuroinflammation (Qian et al., 2022), venous disruption (Zhang et al., 2021) or other extracellular processes - recent studies have highlighted its utility as an indirect marker of ISF accumulation and impaired glymphatic clearance in CSVD (Mayer et al., 2022; Li et al., 2024; Rudilosso et al., 2025). In particular, prior work has demonstrated a robust association between increased FW and WMH severity (Duering et al., 2018; Huang et al., 2022; Lan et al., 2022), reinforcing the role of extracellular water in the pathogenesis of white matter injury.

Despite these findings, two questions remain incompletely understood: (1) topographical differences in PVS-WMH relationships, and (2) the potential mediating role of ISF dynamics quantified by DTI free water. Therefore, this study aims to utilize DTI-based FW analysis to explore its role in PVS-mediated WMH severity.

## 2 Methods

### 2.1 Participants

We collected clinical and neuroimaging data from patients with cerebral small vessel disease who underwent brain MRI at our institution between January and October 2022. Inclusion criteria: age > 40 years; presence of typical CSVD imaging markers on MRI in accordance with the STRIVE (Duering et al., 2023) (e.g., WMH, lacunes, or PVS); at least one vascular risk factor (e.g., hypertension, diabetes, hyperlipidemia, or current smoking). Exclusion criteria: structural brain lesions (e.g., trauma, tumors, hemorrhage, vascular malformations, or acute cerebral infarction), severe stenosis or occlusion of intracranial/carotid arteries (confirmed by vascular ultrasound, magnetic resonance angiography, or computed tomography angiography), other intracranial pathologies (e.g., inflammatory or demyelinating diseases), excessive imaging artifacts affecting diagnostic quality, severe organ dysfunction (cardiac, pulmonary, or renal failure).

### 2.2 MRI acquisition protocol

All participants underwent multi-modal MRI examinations using a 1.5 T Siemens MAGNETOM Aera scanner. The imaging protocol included: 3D T1-weighted imaging (T1w), T2-weighted imaging (T2w), T2 fluid-attenuated inversion recovery (FLAIR), time-of-flight magnetic resonance angiography and DTI. Detailed acquisition parameters: (1) T2w: repetition time (TR)/echo time (TE) = 3800/88 ms, slice thickness = 5 mm, matrix size = 256 × 256, field of view (FOV) = 24 × 24 cm^2^; (2) T2 FLAIR: TR/TE = 7,000/94 ms, slice thickness = 5 mm, FOV = 24 × 24 cm^2^, matrix = 256 × 256; (3) 3D T1w: TR/TE = 2,000/2.84 ms, sagittal slices = 144, slice thickness = 1 mm, matrix = 256 × 256; (4) DTI: TR/TE = 3,600/95 ms, slice thickness = 3 mm, matrix = 128 × 128, diffusion directions = 30 per *b*-value, *b*-values = 0, 1,000, and 2,000 s/mm^2^.

### 2.3 Image analysis

#### 2.3.1 Perivascular space rating

For PVS quantification, we employed a standardized visual rating scale based on established criteria (Duering et al., 2023). The assessment protocol involved: (1) identification of the axial slice demonstrating the highest BG PVS and CS PVS burden regions; (2) counting PVS on the side showing the greatest involvement within each region. The 5-point scoring system was applied as follows: Grade 0 (no visible dilated PVS); Grade 1 (<10 dilated PVS); Grade 2 (10–20 dilated PVS); Grade 3 (21–40 dilated PVS); and Grade 4 (>40 dilated PVS). Cases with BG PVS or CS PVS scores exceeding 1 were classified as high-grade BG PVS or CS PVS respectively, indicating clinically significant PVS enlargement. This threshold is consistent with the criteria used in the total CSVD MRI score (Staals et al., 2014), in which a PVS score >1 contributes one point to the total CSVD burden and reflects moderate-to-severe PVS burden.

PVS ratings were performed on axial T2-weighted images by two experienced neuroradiologists who were blinded to all clinical data and study hypotheses. Inter-rater reliability indicated excellent agreement (*κ* = 0.914 for BG PVS; κ = 0.894 for CS PVS), with discrepancies resolved by consensus.

#### 2.3.2 WMH, normal-appearing white matter and intracranial segmentation

White matter and intracranial volume were automatically segmented from 3D T1w images using FSL’s fsl_anat pipeline (FSL v6.0.7), generating corresponding binary masks for subsequent analyses. T2-FLAIR-based WMH segmentation was performed manually in ITK-SNAP by trained raters. Total WMH volume was calculated, normalized to intracranial volume to account for interindividual differences in head size, and log10-transformed to improve normality for statistical analysis.

To ensure spatial consistency across modalities, the white matter mask, 3D T1w image, and T2-FLAIR image were linearly co-registered to the b = 0 s/mm^2^ volume of the DTI dataset using FSL’s FLIRT tool. Following registration, the WMH mask was subtracted from the white matter mask to generate a normal-appearing white matter (NAWM) mask, which was used for subsequent FW extraction.

#### 2.3.3 FW in normal-appearing white matter

**Figure 1:**
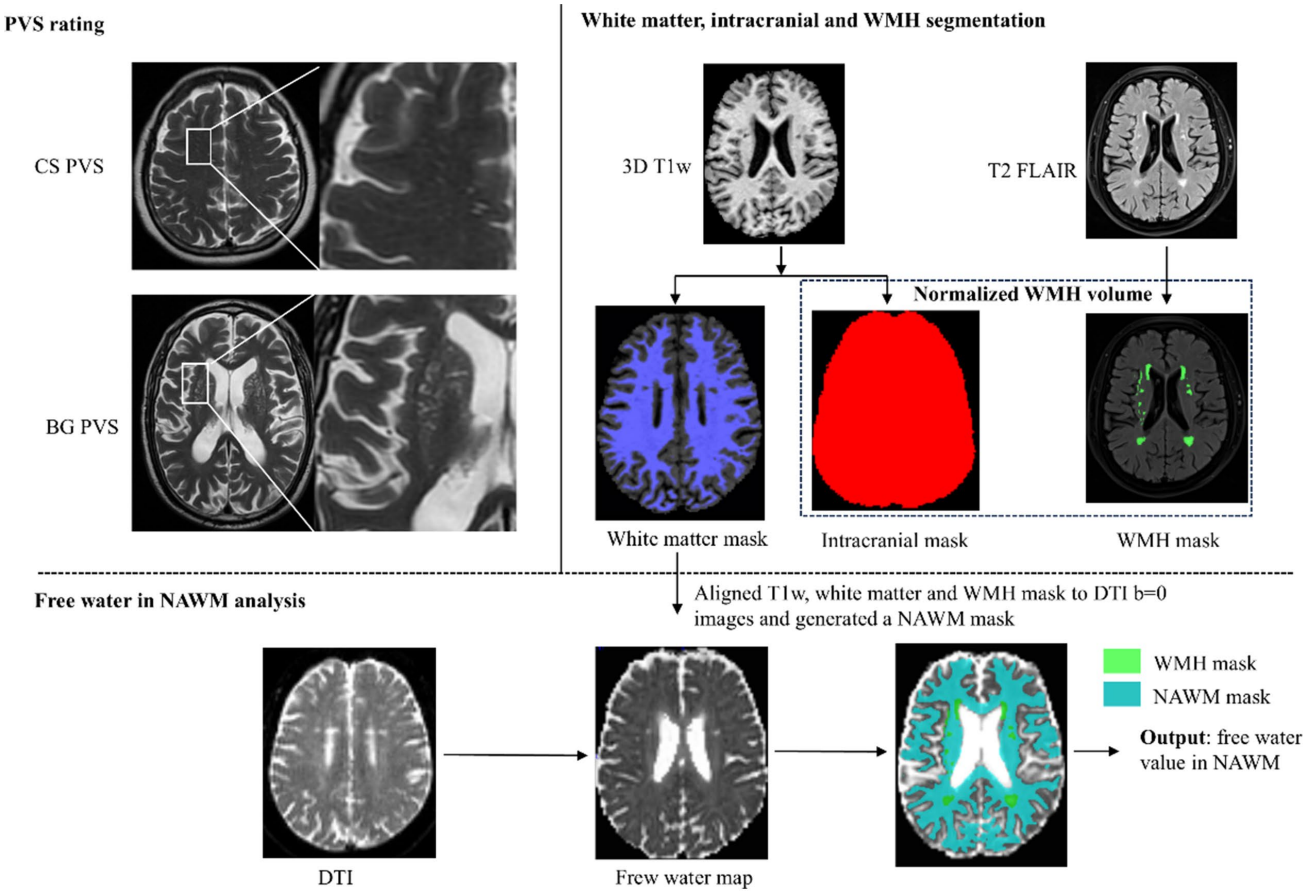
Illustration of the image-processing workflow. PVS, perivascular space; BG PVS, PVS in basal ganglia; CS PVS, PVS in centrum semiovale; DTI, diffusion tensor imaging; WMH, white matter hyperintensity; T1w, T1-weighted imaging; T2 FLAIR, T2 fluid-attenuated inversion recovery; NAWM, normal-appearing white matter.

DTI data were preprocessed using MRtrix3 (version 3.0.2). Preprocessing included Gibbs ringing artifact correction, noise reduction, and correction for echo-planar imaging distortions and eddy currents. To ensure compatibility with the Gaussian assumptions of the bi-tensor model, only the b = 0 and b = 1,000 s/mm^2^ volumes were used for FW estimation, and the b = 2,000 s/mm^2^ shell was excluded. The FW fraction was then estimated using a two-compartment model (Pasternak et al., 2009) implemented in DIPY (Diffusion Imaging in Python), producing FW maps with values ranging from 0 to 1, where higher values reflect increased ISF free water content. Finally, FW values were extracted from voxels within the NAWM mask defined in DTI space. To minimize partial volume effects from adjacent cerebral spinal fluid, particularly near the ventricles, voxels with FW values exceeding 0.70 were excluded from the analysis. The illustration of the image-processing workflow was shown in Figure 1.

### 2.4 Statistical analysis

Continuous normally distributed variables are reported as mean ± SD, non-normal variables as median (IQR), and categorical variables as counts (percentages). For group comparisons, independent samples t-tests or Wilcoxon rank-sum tests were used for FW and normalized WMH volume between groups according to presence of high-grade BG PVS (CS PVS). The relationship between FW and normalized WMH volume was assessed using Pearson’s correlation analysis.

Multivariate linear regression models were constructed to examine: (1) the association between high grade BG PVS (CS PVS) and both FW and normalized WMH volume, and (2) the correlation between FW and WMH volume, with adjustment for potential confounding factors. Mediation analysis assessed whether FW mediated the relationship between high grade BG PVS and normalized WMH volume, using the PROCESS macro (Model 4) with 5,000 bootstrap samples to estimate indirect effects. SPSS 20.0 was used for all analyses, with two-tailed *p* < 0.05 defining significance.

## 3 Results

### 3.1 Baseline characteristics

The study included 125 CSVD patients (mean age 60 ± 11 years; 57 males [45.6%]) with the following vascular risk profile: hypertension (*n* = 67, 53.6%), diabetes (*n* = 22, 17.6%), current smoking (*n* = 27, 21.6%), and dyslipidemia (*n* = 31, 24.8%). High grade BG PVS and CS PVS were observed in 23 (18.4%) and 51 (40.8%) patients, respectively. The cohort demonstrated a mean NAWM FW value of 0.30 ± 0.01 and mean log10 normalized WMH volume of −2.21 ± 0.34.

### 3.2 Association between high grade BG PVS and normalized WMH volume

**Figure 2:**
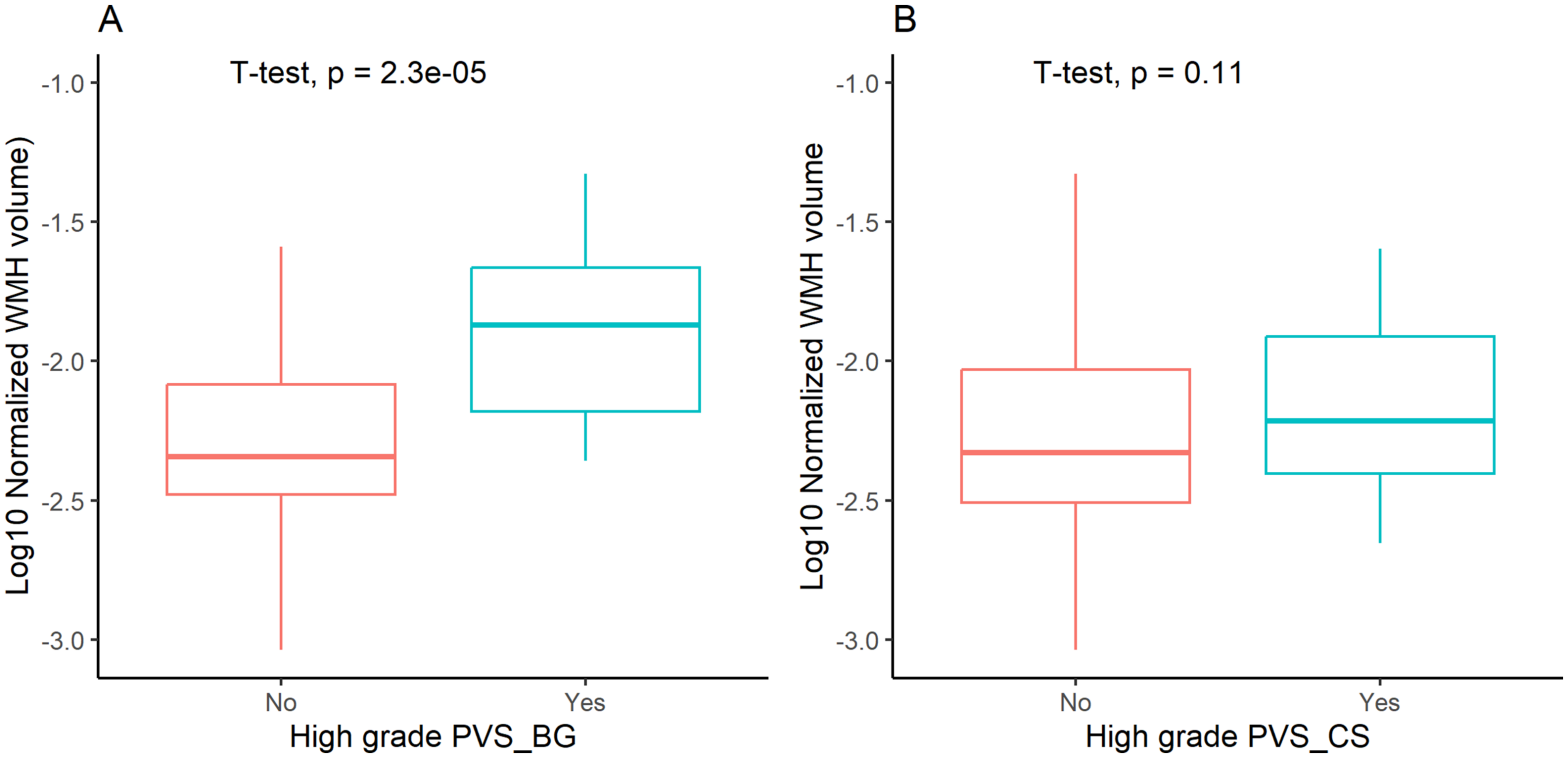
White matter hyperintensity (WMH) volume stratified by high-grade perivascular spaces (PVS). BG PVS, PVS in basal ganglia; CS PVS, PVS in centrum semiovale.

Comparative analysis revealed significantly higher normalized WMH volumes in patients with high grade BG PVS versus those without (*p* < 0.05, Figure 2A), while no significant difference was observed for high grade CS PVS (*p* > 0.05, Figure 2B). Multivariable linear regression adjusted for age and vascular risk factors confirmed an independent association between high grade BG PVS and log10 normalized WMH volume (β = 0.217, 95% CI: 0.041–0.393; *p* < 0.05, Table 1).

**Table 1:** Multivariable analysis of the associations between high grade BG PVS/CS PVS and WMH volume.

Variable	β	*P* value	95.0% CI	
			Lower bound	Upper bound
High grade BG PVS	0.217	0.016	0.041	0.393
High grade CS PVS	0.031	0.706	−0.133	0.195
Age	0.433	<0.001	0.235	0.631
Gender	0.040	0.679	−0.150	0.229
Hypertension	−0.015	0.864	−0.186	0.156
Diabetes mellitus	−0.055	0.505	−0.217	0.107
Current smoking	−0.189	0.047	−0.375	−0.003
Hyperlipidemia	0.050	0.542	−0.111	0.211

Perivascular space in basal ganglia (BG PVS)/centrum semiovale (CS PVS).

### 3.3 Relationship between high grade BG PVS and FW

**Figure 3:**
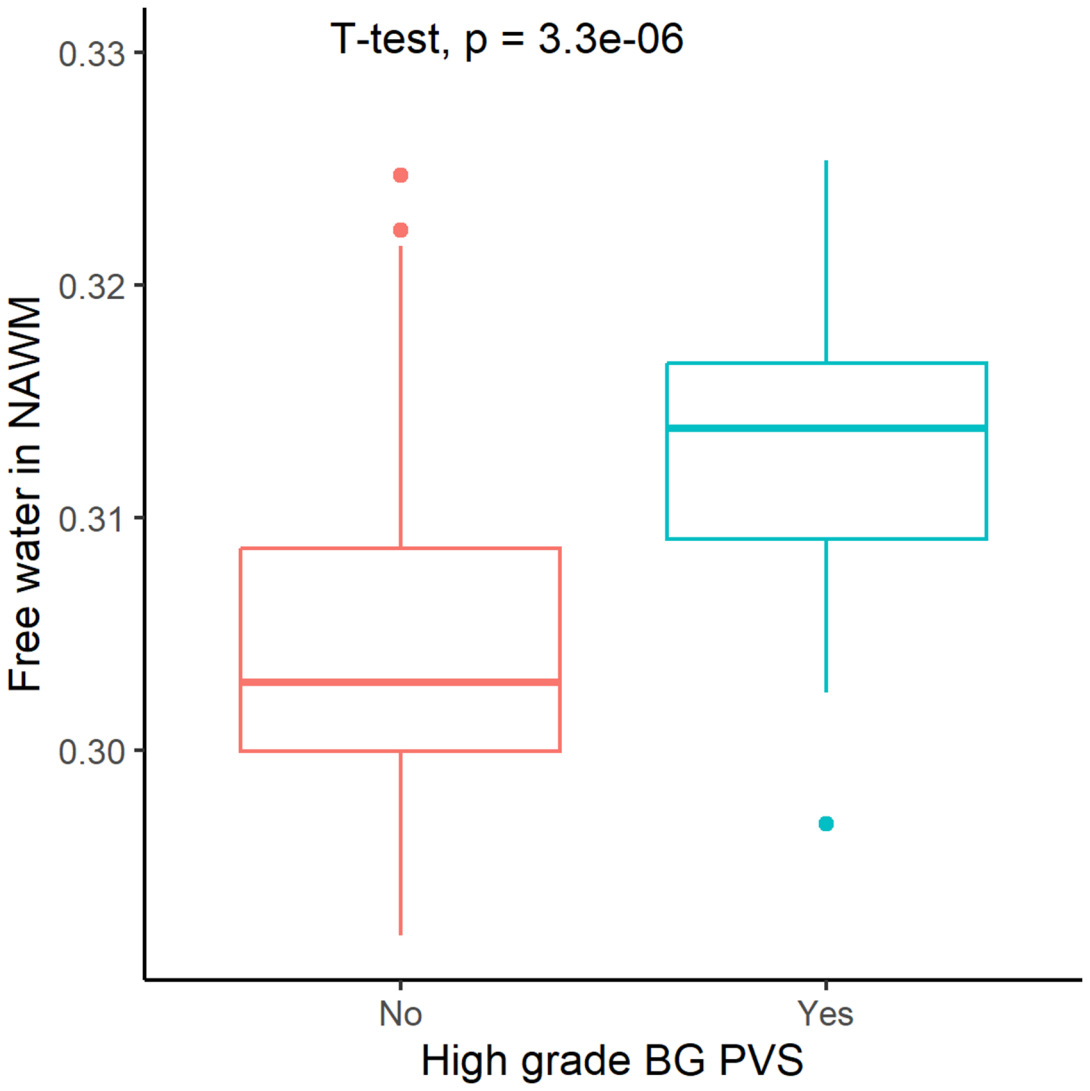
Free water in normal-appearing white matter (NAWM) stratified by high-grade perivascular spaces basal ganglia (BG PVS).

The high-grade BG PVS group exhibited significantly elevated FW values in NAWM compared to group of absence of high-grade BG PVS (*p* < 0.05, Figure 3). This association remained significant after covariate adjustment (β = 0.264, 95% CI: 0.109–0.419; *p* < 0.05, Table 2).

**Table 2:** Multivariable analysis of the associations between high grade BG PVS and free water in NAWM.

Variable	β	*P* value	95.0% CI	
			Lower bound	Upper bound
High grade BG PVS	0.264	0.001	0.109	0.419
Age	0.491	<0.001	0.321	0.661
Gender	0.074	0.369	−0.088	0.236
Hypertension	−0.065	0.391	−0.216	0.085
Diabetes mellitus	0.150	0.040	0.007	0.292
Current smoking	0.000	0.998	−0.162	0.163
Hyperlipidemia	−0.156	0.031	−0.297	−0.015

Perivascular space in basal ganglia (BG PVS); normal-appearing white matter (NAWM).

### 3.4 Correlation between normal-appearing white matter FW and normalized WMH volume

**Figure 4:**
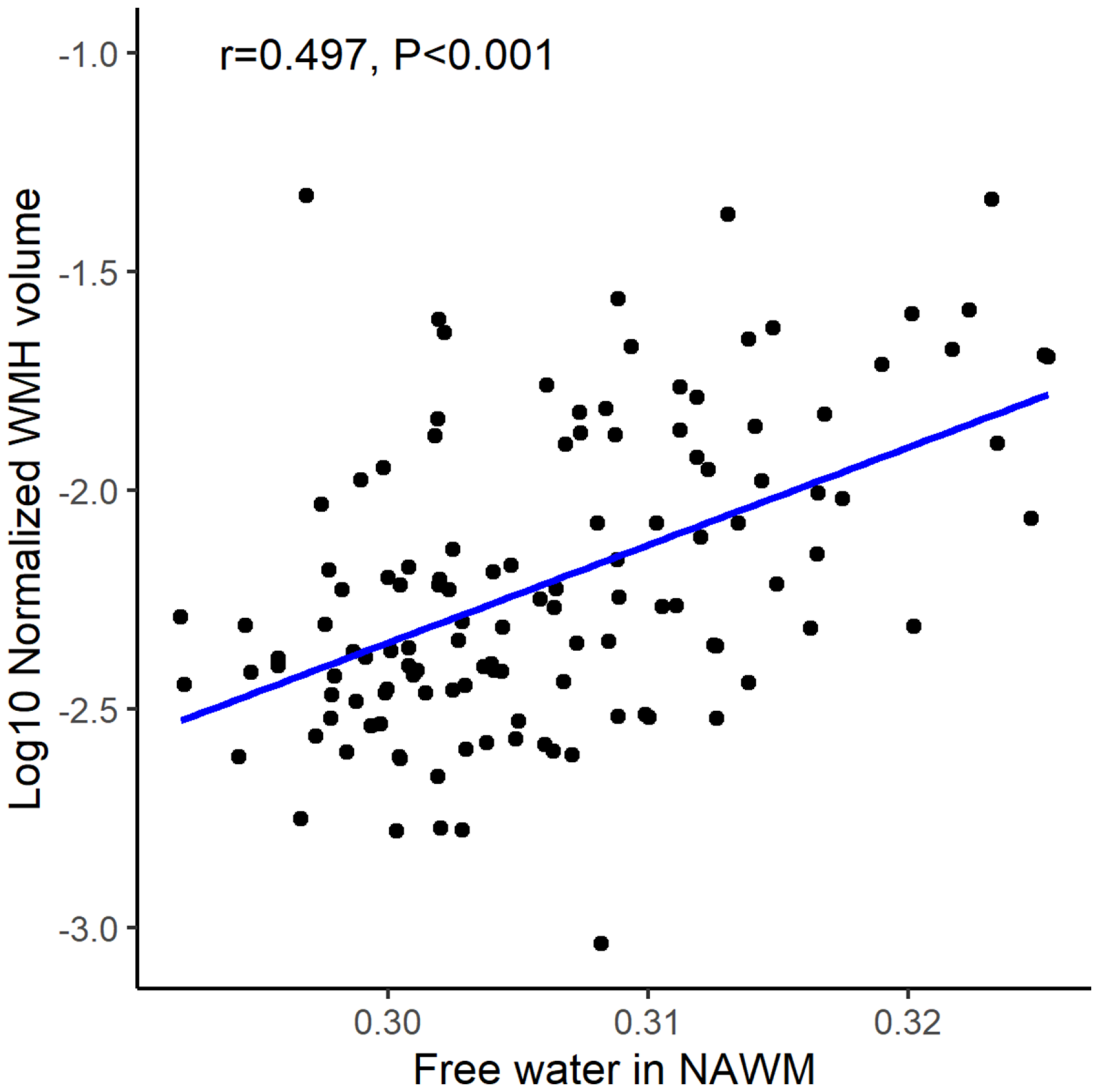
The association of free water in normal-appearing white matter (NAWM) and white matter hyperintensity (WMH) volume.

Pearson’s correlation analysis demonstrated a strong positive correlation between FW values in NAWM and normalized WMH volume (*r* = 0.497, *p* < 0.05; Figure 4). The multivariable model confirmed FW as an independent predictor of WMH burden after adjusting for age and vascular risk factors (β = 0.356, 95% CI: 0.165–0.548; *p* < 0.05, Table 3).

**Table 3:** Multivariable analysis of the associations between free water in normal-appearing white matter and WMH volume.

Variable	β	*P* value	95.0% CI	
			Lower bound	Upper bound
Free water	0.356	<0.001	0.165	0.548
Age	0.326	0.002	0.120	0.531
Gender	−0.007	0.941	−0.184	0.170
Hypertension	0.013	0.872	−0.152	0.179
Diabetes mellitus	−0.130	0.099	−0.284	0.025
Current smoking	−0.174	0.055	−0.352	0.004
Hyperlipidemia	0.119	0.133	−0.037	0.275

### 3.5 Mediation analysis of potential pathways

**Figure 5:**
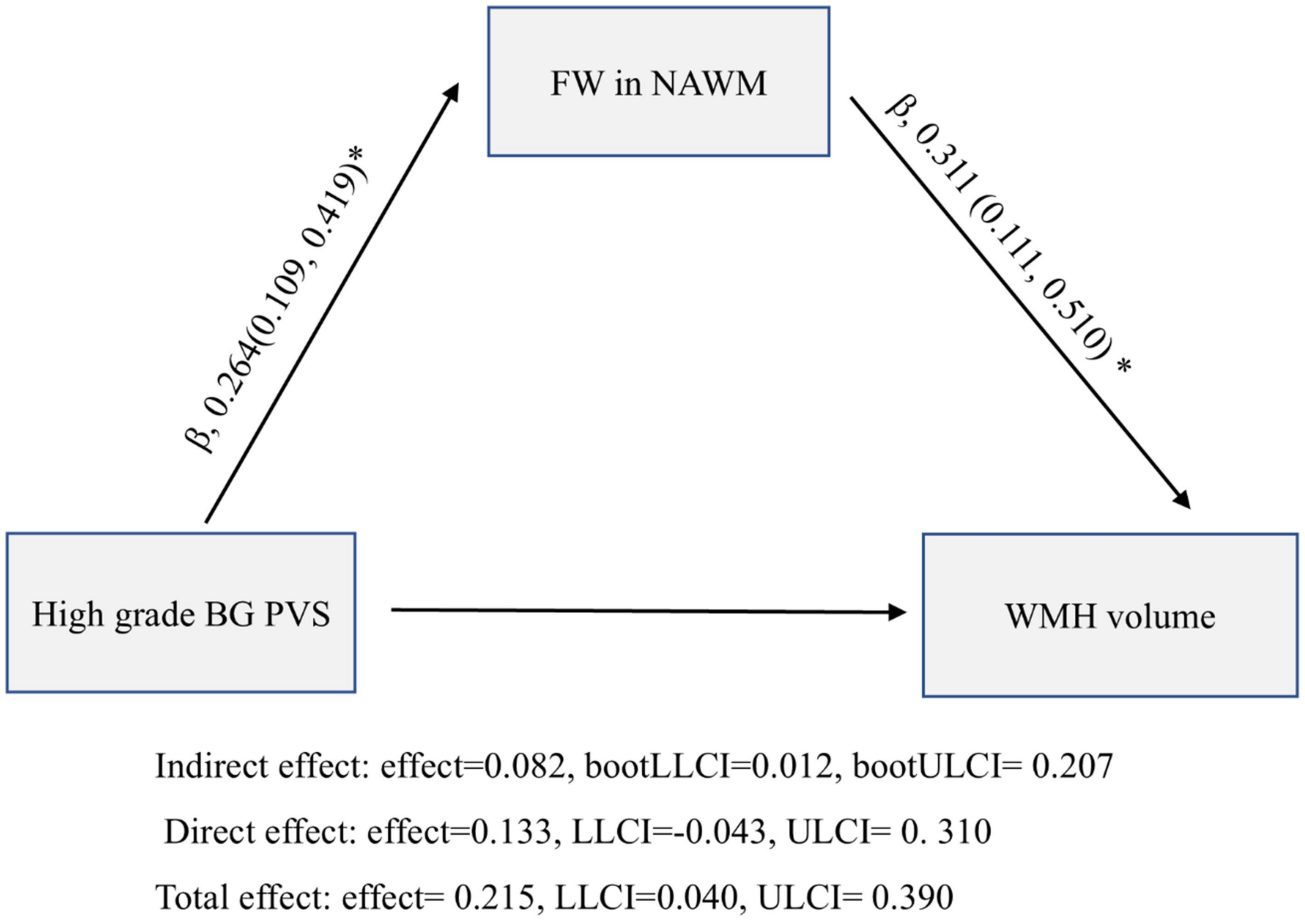
Mediation analysis of the relationship between high grade basal ganglia perivascular spaces (BG PVS), normal-appearing white matter (NAWM) free water (FW), and white matter hyperintensity (WMH) volume.

The mediation model incorporating age and vascular risk factors revealed a significant indirect effect of FW in NAWM on the high-grade BG PVS-WMH association (β = 0.082, 95% CI: 0.012–0.207; *p* < 0.05; Figure 5), accounting for 38.1% of the total effect.

## 4 Discussion

This multimodal neuroimaging study investigated the differential relationships between PVS in distinct anatomical locations and WMH volume. Key findings demonstrated that BG PVS independently correlated with WMH burden, whereas CS PVS showed no significant association. Mediation analysis revealed high grade BG PVS were associated with WMH volume partly through elevated FW fraction, suggesting FW may link BG PVS dilation to white matter damage.

On T2-weighted imaging, PVS represent critical anatomical components of the glymphatic system surrounding cerebral arteries (Oltmer et al., 2024), facilitating ISF drainage and metabolic waste clearance (Wardlaw et al., 2020). Arterial pulsation may serve as a key driving force for fluid movement within PVS. Experimental studies have demonstrated a positive correlation between arterial pulsation amplitude and PVS flow velocity, where diminished pulsation reduces PVS flow rates (Dreyer et al., 2024). The mechanism by which attenuated arterial pulsation may lead to PVS dilation involves multiple interrelated factors spanning biomechanical, molecular, and hydrodynamic domains: (1) reduced pulsation elevates hydrostatic pressure within PVS, potentially causing structural expansion. Hsu et al. reported a significant correlation between PVS dilation and decreased DTI-ALPS index (Hsu et al., 2024). (2) impaired arterial pulsation may secondarily affect cerebral venous outflow, compromising cerebrospinal fluid reabsorption and exacerbating PVS dilation. This is supported by Zhang et al.’s findings demonstrating associations between deep medullary vein disruption and PVS enlargement (Zhang K. et al., 2022). (3) astrocytic AQP4 regulation: The polarization of aquaporin-4 channels on astrocyte end-feet depends on mechanical stresses generated by arterial pulsations to modulate ISF-CSF exchange. Pulsation attenuation can disrupt AQP4 polarity, impairing glymphatic function (Gomolka et al., 2023).

We found that high-grade BG PVS, but not CS PVS, independently correlates with WMH volume, underscoring the topographical heterogeneity of PVS pathophysiology. This anatomical specificity may stem from distinct vascular origins. BG PVS originate from lenticulostriate arteries - high-pressure vessels branching from middle cerebral arteries with strong pulsatile forces (van den Kerkhof et al., 2023). Their dilation reflects hypertensive arteriopathy/arteriosclerosis (Duperron et al., 2023), where excessive pulsatility may impair periarterial drainage (Rudilosso et al., 2025). Hypertension-induced dampened arterial waves could compromise ISF clearance, ultimately contributing to WMH. Importantly, our mediation analysis suggested that increased ISF content, as estimated by FW, partially mediated the association between BG PVS burden and WMH volume. This supports the notion that glymphatic dysfunction and impaired interstitial clearance may serve as intermediary processes linking perivascular alterations to downstream white matter damage in CSVD. While FW is not specific to any single biological mechanism, it offers a noninvasive proxy for extracellular fluid burden and provides valuable insight into fluid-related pathways of tissue injury. This interpretation is consistent with recent longitudinal findings by Kern et al. (2023) who reported that elevated FW predicted the progression of NAWM to WMH over 1 year follow-up, further supporting the role of interstitial fluid accumulation as an early marker of white matter vulnerability in CSVD.

In contrast, CS PVS derive from superficial cortical arterioles with lower flow velocity and pulsatility (van den Kerkhof et al., 2023). Their unbranched trajectories rarely penetrate deep into subarachnoid spaces, suggesting primary involvement in cortical metabolic clearance rather than high-pressure interstitial drainage. The lack of independent CS PVS-WMH association may imply minimal contribution to interstitial edema-related white matter damage. Existing evidence instead implicates CS PVS in cerebral amyloid angiopathy, particularly β-amyloid deposition around cortical/leptomeningeal vessels causing vascular stiffening and perivascular expansion (Tsai et al., 2021). PET-CT studies confirm CS PVS correlates strongly with amyloid burden and lobar microbleed risk (Charidimou et al., 2017; Kim et al., 2021).

Notably, some cases exhibited severe WMH despite mild BG PVS dilation, underscoring WMH’s multifactorial etiology [e.g., hypoperfusion (Zhang R. et al., 2022), deep venous disruption (Xu et al., 2020; Zhang et al., 2021), blood–brain barrier leakage (Wong et al., 2019; Li et al., 2023)]. Multimodal neuroimaging techniques may enable more comprehensive exploration of the mechanisms underlying WMH formation and progression in future studies, including investigations into how various pathogenic factors interact with each other.

Study limitations include: (1) single-center design with limited sample size. The high grade PVS-BG group had a smaller sample size and greater variance, which may increase the likelihood of type I error or overestimated associations. Therefore, the finding that BG PVS was significantly associated with WMH burden, while CS PVS was not, should be interpreted with caution. Future studies with larger and more balanced subgroup sizes are warranted to confirm the topographical specificity of these relationships. (2) Use of visual rating for PVS assessment. While widely used in CSVD research, visual PVS grading may be less precise than fully automated segmentation. Although we attempted to apply publicly available deep learning–based tools for 3D T1-weighted images, their performance was suboptimal in our dataset—particularly in deep brain regions. We therefore used a validated visual scale on axial T2-weighted images, which showed excellent inter-rater agreement (*κ* = 0.914 for BG PVS; κ = 0.894 for CS PVS). Future studies using robust automated segmentation approaches may enhance reproducibility and sensitivity. (3) MRI acquisition on 1.5 T scanner. Diffusion tensor imaging data for FW estimation were acquired at 1.5 T with 30 directions and 3 mm isotropic voxels. Compared to higher-field scanners (e.g., 3 T or 7 T) and advanced protocols (e.g., 64 + directions, <2 mm resolution), these settings may offer lower SNR and spatial resolution, possibly affecting FW precision. Although FW modeling has been validated under similar conditions (Duering et al., 2018), future replication with high-resolution, multi-shell acquisitions is warranted. (4) unaccounted FW confounders, such as neuroinflammation, venous hypertension; (5) lack of longitudinal PVS-FW-WMH trajectory analysis. Future multi-center studies incorporating molecular imaging and perfusion metrics should elucidate PVS’s location-specific roles across cerebral small vessel disease subtypes and their biomarker potential.

In summary, PVS topography encodes distinct small vessel disease mechanisms. The association between BG PVS, free water accumulation, and WMH volume suggests pulsatility-related ISF dysfunction may underlie this linkage—a hypothesis warranting prospective investigation to inform therapeutic targeting. Future studies should integrate proteomic and transcriptomic data with multimodal imaging to fully elucidate these molecular pathways.

## Data Availability

The original contributions presented in the study are included in the article/supplementary material, further inquiries can be directed to the corresponding author.
